# Supplementary material for: The concordance of signals based on irregular incremental lines in the human tooth cementum with documented pregnancies: Results from a systematic approach
Source: PLoS One. 2022 Sep 9;17(9):e0267336. doi: 10.1371/journal.pone.0267336 (PMC9462792; doi:10.1371/journal.pone.0267336)
Supplement: S1 Text — (PDF) [file pone.0267336.s001.pdf]

S1 Text. Source research for the reference skeleton series „Basel-Spitalfriedhof".

In 1988 and 1989, parts of the former cemetery of the Bürgerspital Basel were excavated in the course of an emergency archaeological excavation. Between 1845 and 1868 a total of 2,561 patients from the Bürgerspital were buried in this hospital cemetery. In 1988/89 over 1,000 graves were exhumed. In 1990, because the burials were carried out in chronological order, 80% of the skeletons could be identified with the help of the register of graves (StABS, Bau JJ 44.), the register of deaths in the Bürgerspital (StABS, Spital AA 2.4) and the anthropological age and sex determination of the exhumed skeletons. This allowed each skeleton to be assigned a name, age, origin, profession and illness.

Since 2007, within a large Citizen Science Project (Citizen Science Basel, CSB) historical files related to the skeletons of the Basel-Spitalfriedhof have been transcribed in order to create a basis for scientific projects. The files can be found mainly in the State Archives Basel City (StABS). In a first step, all multi-page medical files belonging to a skeleton were transcribed. Nearly 1,000 transcribed medical records allowed the identification carried out in 1990 to be verified. For example, the operation descriptions noted in the medical records can be matched with the operation traces found on the skeletons.

In addition to the medical files, patient registers, medical annual reports, and other serial records were also transcribed. At present, there is over 7 million historical personal data available in digital form for the skeletons, which enables the reconstruction of the living conditions of these individuals. The identified skeletons of the Basel-Spitalfriedhof can be considered one of the best documented anthropological reference series worldwide.

The basis of the present investigation is the death register of the Bürgerspital (StABS Spital AA 2.4), which records all patients who died in the hospital with their name, age, profession, marital status, illness, origin, and date of death. In some cases, the birth dates of the deceased person were also recorded. This personal information serves to identify the historical persons in other sources, such as the birth register. The procedure is shown graphically in Fig. S1a. The age recorded in the register of deaths was verified by finding the date of birth in the corresponding birth and baptism registers. Here, baptismal registers from the county of Basel as well as neighbouring regions had to be consulted to verify the data. At the same time, the number of births for each individual woman were researched from the birth registers. This was a complex undertaking, as the registers were written down in a Kurrent script that was difficult for us to decipher.

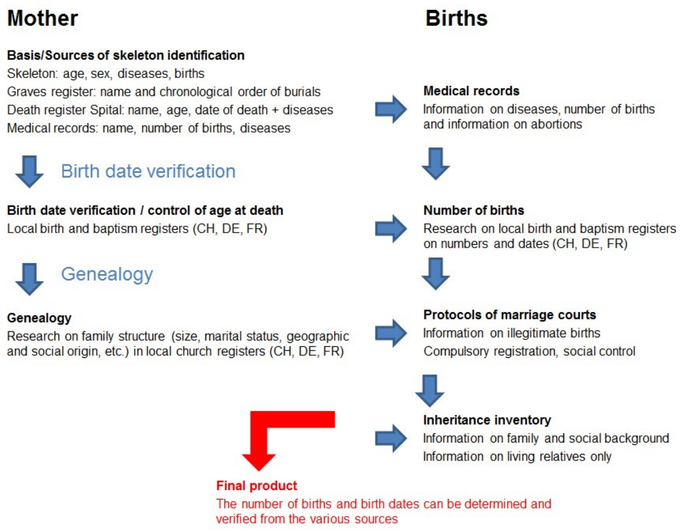

Fig. S1 a: Flow chart of the historical file research of information about the women and births

Also fundamental, were the medical records of the Bürgerspital (StABS Spital V 30), which usually indicated how many children a woman had given birth to and this information also proved to be reliable as it could be verified in other sources. Similarly, the medical records also contain information on any abortions and stillbirths. The medical records are available for the medical department from the beginning of 1842 and for the surgical department from 1862. The source-related gap from 1842 to 1861 for the surgery of the Bürgerspital has a direct impact, since the obstetrics department was organisationally affiliated to surgery.

Such gaps could be closed with another valuable source, the so-called "Marriage Court Records". Unmarried pregnant women had to report themselves to the authorities until 1873, as an illegitimate pregnancy was a criminal offence. Accordingly, the women and children's fathers were then subjected to an interrogation and the reports were recorded. Thus, we have detailed information on the children born out of wedlock. Of a total of 38 recorded births and abortions considered in the paper, 18 births and three abortions were of illegitimate nature and 17 births were of marital origin.[1]

Another valuable source are the so-called inheritance inventories, which were prepared in the Bürgerspital in the event of a patient's death. As a rule, these inventories list not only the bequeathed property but also the deceased's family circumstances. This gives an insight into the family situation. However, only relatives living at the time of the inventory are listed.

[1] 10 women gave birth to 18 illegitimate children and had three abortions. Six married women gave birth to a total of 17 children. Of the married women, no records of abortions have been passed on. Eight women gave birth to no children.

Table S1 a: Overview of the historical sources used

Historical Sources	Information	Time frame
Death register of the Bürgerspital Basel	Name, age, origin, disease, occupation, date of birth (in most cases), (with birth date verification, see birth register), age-at-death	1842-1868
Medical Record (Dep. of medicine)	Name, age, occupation, origin, disease and treatment of illness, number births (without any further information), information about aborts	1842-1868
Medical Record (Chirurgical Department)	Name, age, occupation, origin, disease and treatment of illness, number of given birth (without any further information), information about aborts	1862-1868
Birth register (different region and countries)	Name, date of birth of the mother and children, name of parents, name of witness	18./19. Century
Marriage register (different region and countries)	Names of the couples, date of marriage, name of parents, name of witness	18./19. Century
Marriage court records	Illegitimate pregnancy, name of the parents, date of birth, name of child	18./19. Century
Inheritance inventories	Name of deceased person, names of relatives, list of possession	18./19. Century

In conclusion, it can be said that the available genealogical data were collected by an experienced team of several genealogists from the CSB project between 2013 to 2019. Some individual cases were excluded from the scientific investigation because gaps in the historical records became apparent. These exclusions are based on years of experience through the Basel-specific sources. For source-critical reasons, in this study, women born in Basel were especially taken into account, as they had an excellent historical source situation. Women from the Baden, Württemberg, or Alsace regions were only taken into account if they had a good source situation. Based on several years of genealogical source research and, above all, on the existence of an excellent source situation for 19th century Basel, we assume that only a very small proportion of pregnancies were not recorded.
