# Supplementary material for: The concordance of signals based on irregular incremental lines in the human tooth cementum with documented pregnancies: Results from a systematic approach
Source: PLoS One. 2022 Sep 9;17(9):e0267336. doi: 10.1371/journal.pone.0267336 (PMC9462792; doi:10.1371/journal.pone.0267336)
Supplement: S2 Text — (PDF) [file pone.0267336.s002.pdf]

**S2 Text. Process to manually identify potential signals.**

Incremental line width growth curves

- The incremental line (IL) width, appearance and quality summary measurements of each tooth section are displayed graphically in the IL width growth curve (Fig. S2 a). The process of the measurement and indexing of the ILs is described in Mani-Caplazi et al. (2019). Each tooth is typically represented by three IL width growth curves corresponding to the three analysed sections. The IL appearance and quality has been assessed in Mani-Caplazi et al. (2019) as an index defined by three levels (0=low, 1=medium, 2=high). Each point of the curve represents the mean width per IL (average of the three IL width measurements per section). Irregular IL width, deviations of 1 SD, 2SD and 3SD from the local mean, were defined as three levels of peaks (1-SD, 2-SD and 3-SD peaks).
- Each mean IL width in the curve is displayed in colour representing the Summary Appearance Index (IAP) (summary of the three Appearance Indices per section):
    - Green: Represents a regular appearing section IL with an IAP≤ 2
    - Orange: Represents a just slightly irregular appearing section IL, brighter or darker as the neighbouring ones, with an IAP of 3 or 4
    - Red: Represents a strongly irregular appearing section IL, brighter or darker as the neighbouring ones, with an IAP ≥ 5
- Each mean IL width in the curve is represented by a symbol showing the Summary Quality Index (IQU) (summary of the three Quality Indices per section):
    - Triangle: Represents a poorly distinguished IL, not clearly visible and difficult to identify section IL with an IQU≤ 2.
    - Rhomboid: Represents a moderately distinguished, identifiable section IL with an IQU of 3 or 4.
    - Quadrat: Represents a clearly distinguished, well identifiable section IL with an IQU ≥5.

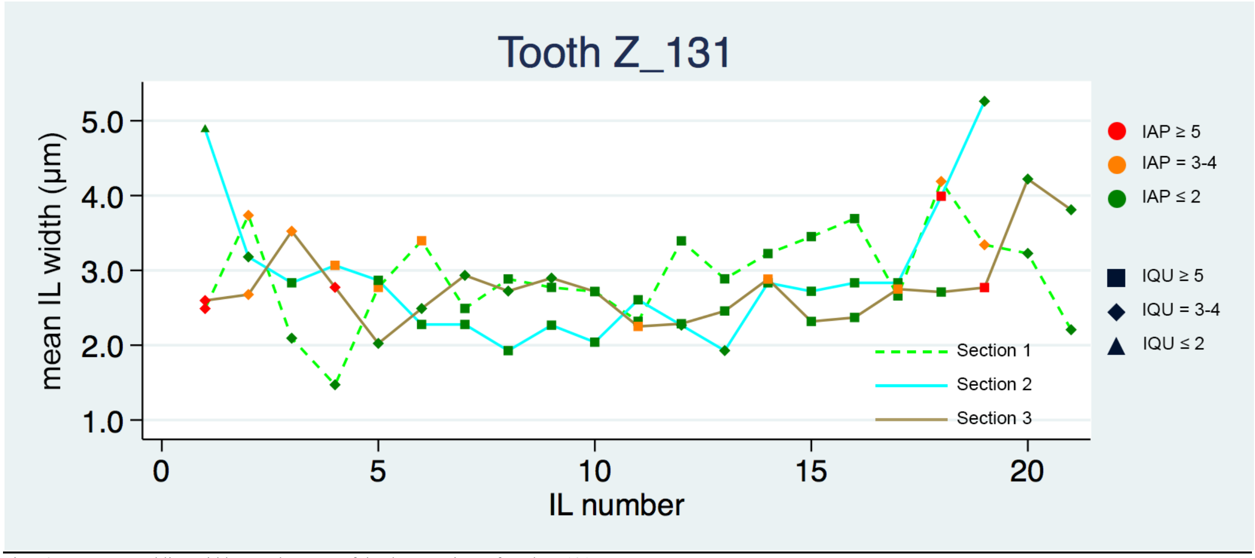


Fig. S2 a: Incremental line width growth curves of the three sections of tooth Z_131

Process to manually identify potential signals based on the incremental line width growth curves and the section images

The following procedure was used to identify irregular ILs based on the IL width growth curves and consulting the corresponding section images. These identified irregular ILs across sections are regarded as potential signals and the term signal is used in the following text. The final output of this process is exemplified in Table S2 a and Table S2 b.

1. Identify and circle areas within the IL width growth curves with irregularities in width, visible as increased amplitudes (potential peaks) and irregular appearances (orange and red spots), only consider irregularities which are present in at least two of three sections or very intensive ones present in only one section (if only two sections were assessed signals in only one section are considered). Due to the IL alignment issue across sections described in Mani-Caplazi et al. (2019) irregular ILs of the same signal may appear in another section at a slightly different IL number. Across section signals can therefore range over several ILs. This first step is only an approximate identification of across section irregularities (Fig. S2 b). In case the number of ILs per section varied, a corresponding plot with reverse numbering of the ILs was also inspected (see in SI 3, Fig. S3 e).

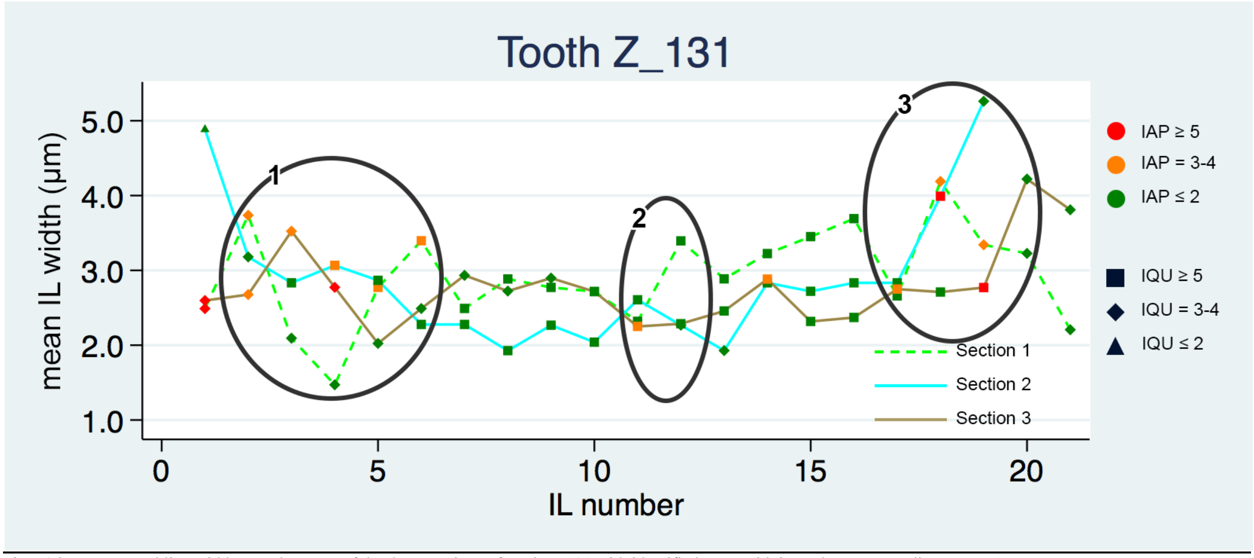


Fig. S2 b: Incremental line width growth curves of the three sections of tooth Z_131 with identified areas with irregular ILs according to step 1.

2. Include the information captured in Fig. S2 b in a list: signal number starting with 1 and the IL numbers of the irregular ILs within this signal.
3. Screen each section image to find the above identified signals and review the signal allocation across sections to ensure corresponding signals across sections are labelled with the same signal number. The aim is to ensure that signals across sections should show a visual similarity. Adjust the IL numbers of the signal accordingly in the excel list if needed for each section. In case a signal can be split into several ones (e.g. sub-signals just separated by few ILs in some, but not necessary all sections), label the signals by additional suffixes a, b, or c. Relabel the corresponding signals of the other sections accordingly.
4. Referring to the example above (Z_131) it is visible on the images that the signal 1 identified in step 1 in the IL width growth curves consists of two signals, which are very close together and have accordingly been captured as signal 1a and 1b (Fig. S2 c, Fig. S2 e; Table: S2 a). The labelling is done similarly as for the usual signals however a, b, c sub-signals are also captured if they are just identified in one section regardless of their intensity. Remove signals identified in Step 1 in case they are considered as too weak. For example, signal 2 refers to a weak potential signal with an increased amplitude only in section 1 (as per peak definition no peak) and a weak appearance signal in section 3 (Fig. S2 a). This signal could not be further substantiated as a signal visible across the three sections in the visual analysis, and did finally not satisfy the rule described in step 1 requiring a signal to be present in at least two sections of three sections or a very intensive signal present in only one section.
5. Add a label describing the identified signal intensity in each section images reviewing the signal intensity along the full image and considering the width (three defined levels of peaks), the IAP and IQU: Intensive signal: ++, less intensive signal: +, faint signal: 0. (Table: S2 a, column: Signal intensity section).
6. Add a label describing the degree of matching of the signal pattern across sections: Very good location matching across sections of very clearly visible signals: ++, Good location matching across sections of clearly visible signals: +, less clear location matching of less well visible signals: 0. (Table: S2 a column: Signal matching across sections).



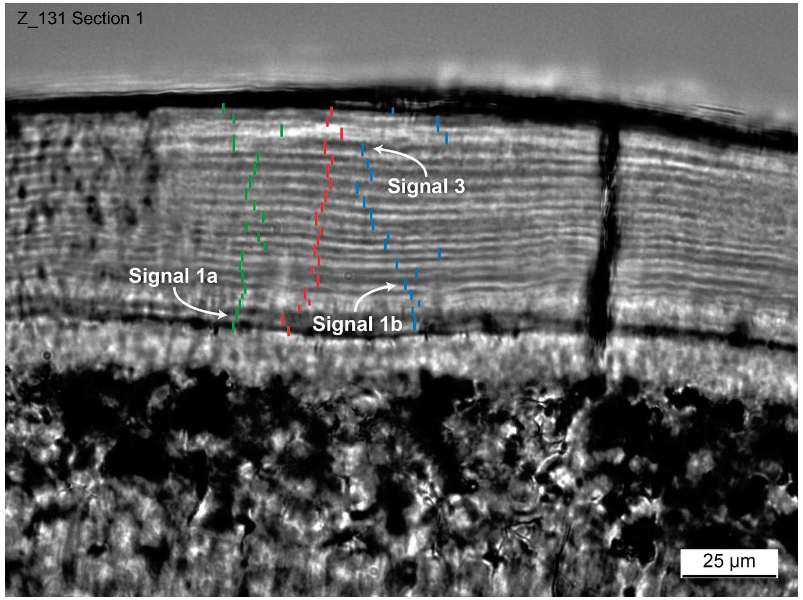


Fig. S2 c: Image of section 1 of tooth Z_131 with identified signals

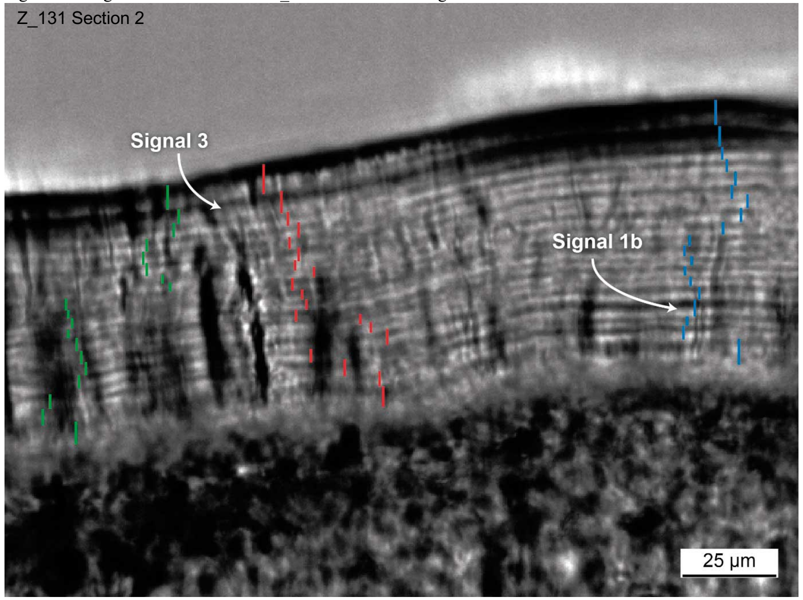


Fig. S2 d: Image of section 2 of tooth Z_131 with identified signals

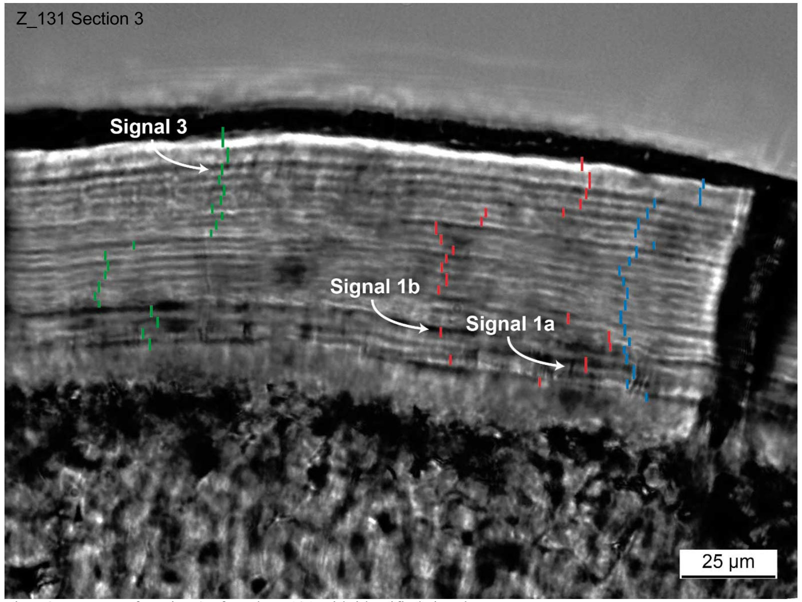


Fig. S2 e: Image of section 2 of tooth Z_131 with identified signals



Table S2 a: Final output of the signal identification based on the incremental line width growth curves (Z_131) and the corresponding section images

| Tooth/ Section number | Image number | Signal number | IL number | Signal intensity section | Signal matching across sections |
|---|---|---|---|---|---|
| Z_131_ Section 1 | R_8251953 | 1a | 2 | + | + |
| | | 1b | 5 | 0 | + |
| | | | 6 | | |
| | | 2 | 18 | + | ++ |
| | | | 19 | | |
| Z_131_ Section 2 | R_95413208 | 1a | none | n/a | n/a |
| | | 1b | 4 | + | + |
| | | 2 | 18 | ++ | ++ |
| Z_131_ Section 3 | R_14498901 | 1a | 2 | + | + |
| | | | 3 | | |
| | | 1b | 4 | ++ | + |
| | | 2 | 19 | + | ++ |
| | | | 20 | | |

**Further example tooth PRG028**

Four clear signals are visible in the IL width growth curves (Fig. S2 f) with signal 1 and 3 containing sub-signals present across sections (Fig. S2 g, Fig. S2 h; Fig. S2 i). The final output is captured in Table S2 b.

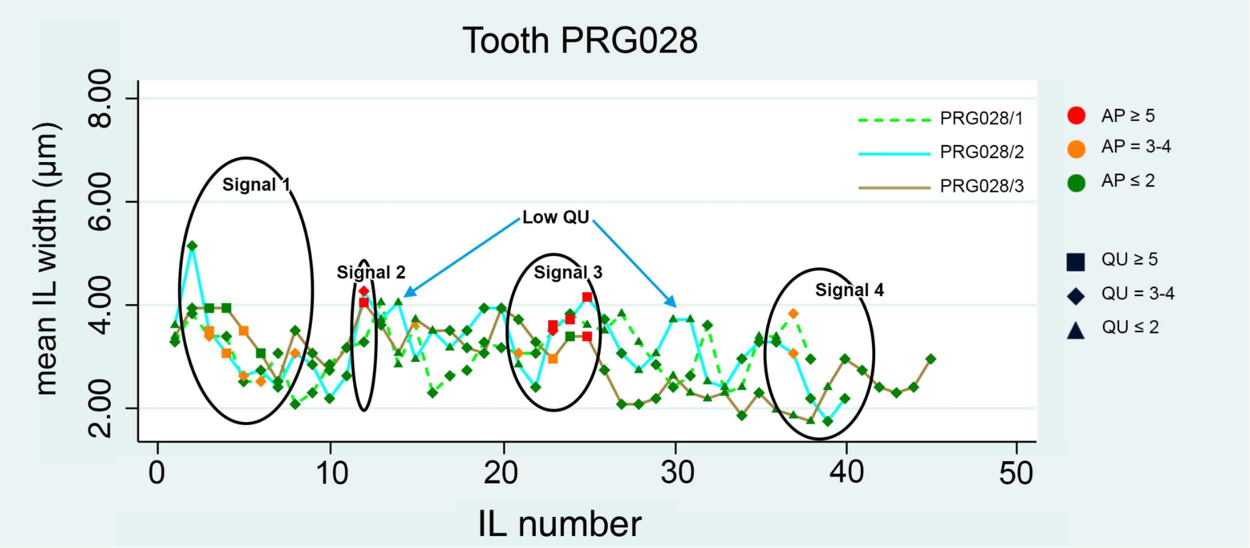


Fig. S2 f: Incremental line width growth curves of the three sections of tooth PRG028 with identified areas with irregular ILs. Two potential peaks (labelled with low QU) which are not well distinguished ILs, providing less reliable IL width measurements were not captured as peaks. Sub-signals were not displayed in the graphic.



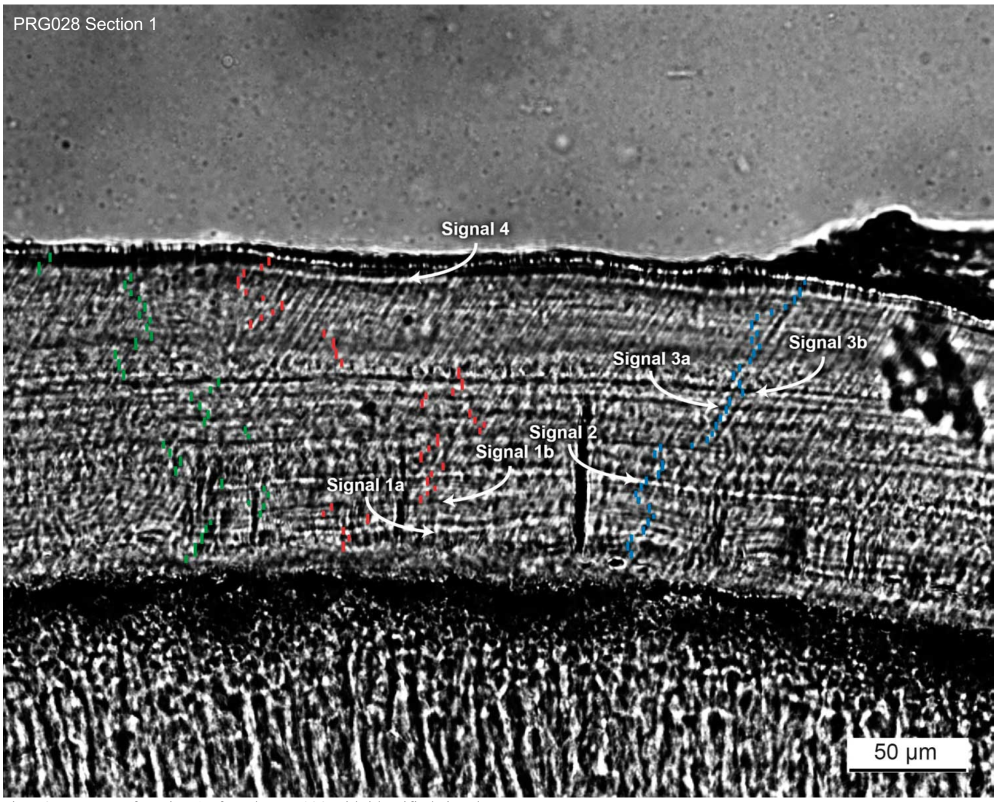


Fig. S2 g: Image of section 1 of tooth PRG028 with identified signals

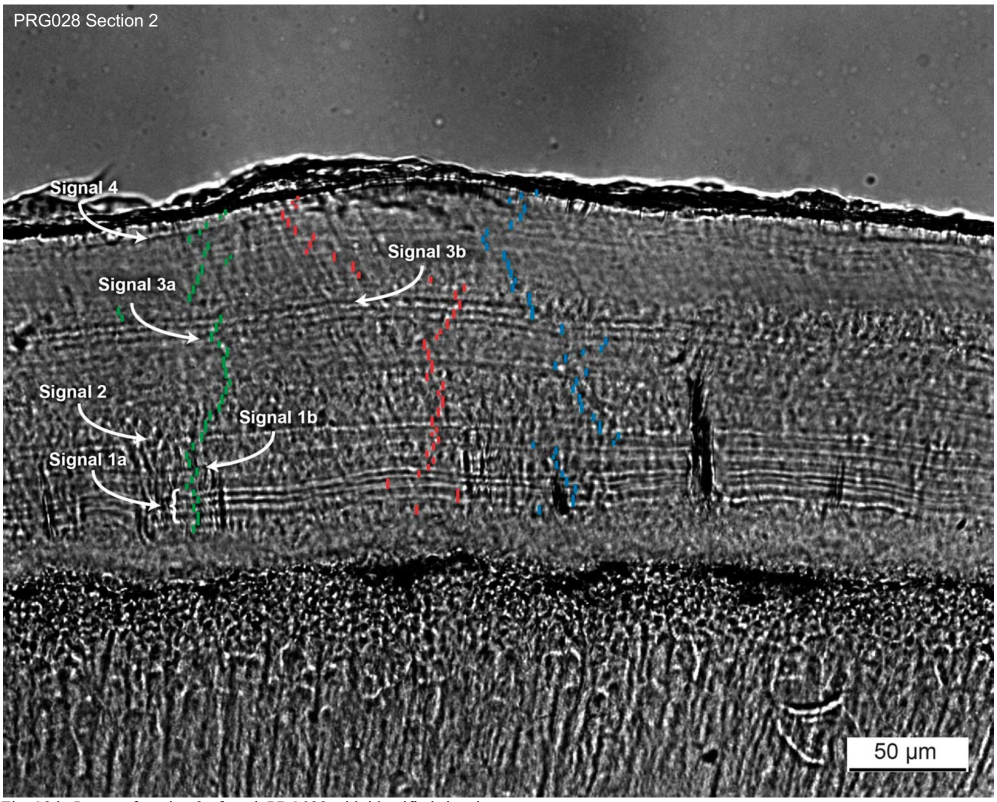

Fig. S2 h: Image of section 2 of tooth PRG028 with identified signals



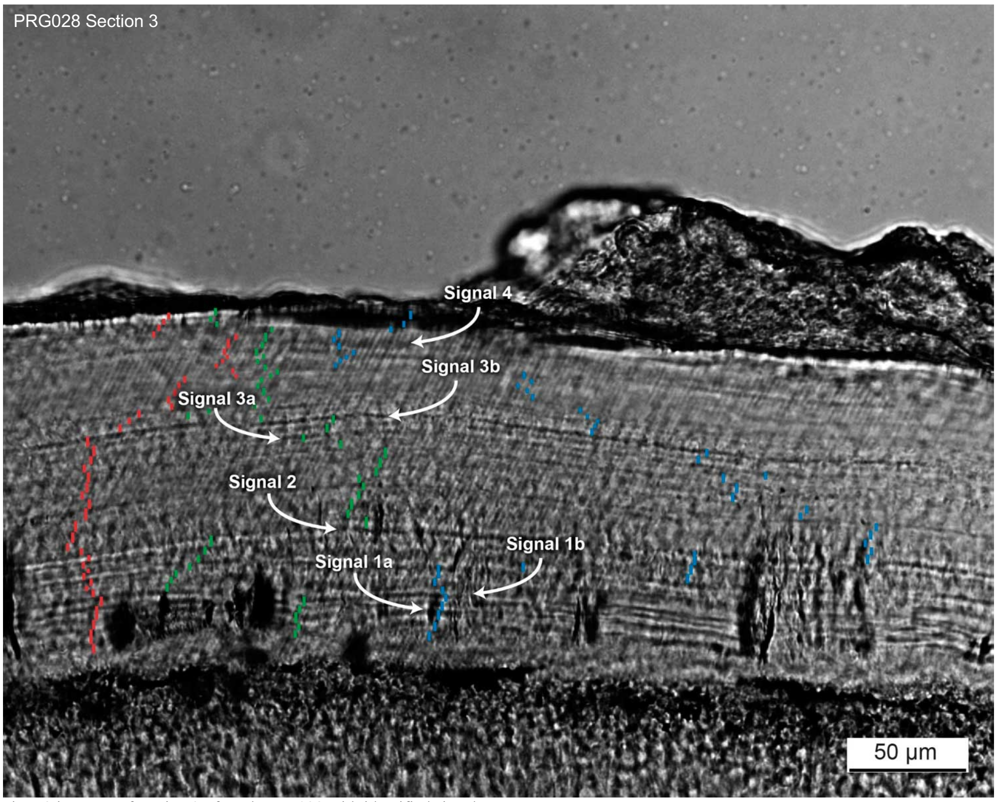

Fig. S2 i: Image of section 3 of tooth PRG028 with identified signals

Table S2 b: Final output of the signal identification based on the incremental line width growth curves (PRG028) and the corresponding section images

| Tooth/ Section number | Image number | Signal number | IL number | Signal intensity section | Signal matching across sections |
|---|---|---|---|---|---|
| PRG028/1 | _PRG028001073 | 1a | 2 | 0 | + |
| | | | 3 | | |
| | | 1b | 6 | 0 | + |
| | | 2 | 11 | + | ++ |
| | | 3a | 21 | ++ | + |
| | | 3b | 23 | ++ | ++ |
| | | 4 | 36 | + | + |
| | | | 37 | | |
| PRG028/2 | _PRG028001075 | 1a | 2 | ++ | + |
| | | | 3 | | |
| | | | 4 | | |
| | | | 5 | | |
| | | 1b | 8 | + | + |
| | | 2 | 12 | ++ | ++ |
| | | 3a | 23 | + | + |
| | | | 24 | | |
| | | 3b | 25 | ++ | ++ |
| | | 4 | 37 | 0 | + |
| PRG028/3 | _PRG028001077 | 1a | 3 | + | + |
| | | 1b | 5 | + | + |
| | | 2 | 12 | ++ | ++ |
| | | 3a | 23 | + | + |
| | | 3b | 25 | ++ | ++ |
| | | 4 | 40 | + | + |
| | | | 41 | | |
