# Supplementary material for: The concordance of signals based on irregular incremental lines in the human tooth cementum with documented pregnancies: Results from a systematic approach
Source: PLoS One. 2022 Sep 9;17(9):e0267336. doi: 10.1371/journal.pone.0267336 (PMC9462792; doi:10.1371/journal.pone.0267336)
Supplement: S3 Text — (PDF) [file pone.0267336.s003.pdf]

S3 Text. Manual assessment of identified signals and overlap with documented pregnancies: Cases.

Case Z_131 (archaeological tooth)

Name: Rosina Drüssel

Birth date: 11.03.1834 documented in the church record books (Kirchenbücher: Sumiswald, Buch 20 – Taufrodel auswärts 1797-1840, pp. 116)

Death date: 20.07.1865 (cause of death documented in the medical record: Typhus) documented in the death register in the state archives (StABS Spital AA 2.3a Sterbe- und Beerdigungsregister 1864-1877) and the inheritance inventories in the state archives (StABS Gerichtsarchiv p. 1.186-187, Erb-Inventare 1865, No. 243, Rosine Drüssel).

Documented pregnancy, abortion and one other life event

- Childbirth: 10.11.1863 (illegitimate pregnancy). The pregnancy was documented in the following court records in the state archives (StABS Gerichtsarchiv U 156, 7.6.1862-13.6.1864, Ehegerichtsprotokoll vom 06.07.1863). The childbirth was documented in the baptismal- and birth register archived in the state archives (StABS Spital AA 1.4 Geburts- und Taufregister 1842-1876).
- Abortion (4th month of pregnancy): 19.07.1865 (illegitimate pregnancy). The pregnancy and abortion were documented in the medical record in the state archives (StABS Spital V 30.27, Fol. 726).
- Death of Rosina Drüssel’s mother Anna Elisabeth Wullschlegel: 02.12.1845. The death is documented in the death register (Billeter-Sammlung, GHGRB Basel, unter Trüssel, Sumiswald, p. 7). This type of event is not considered in the overall research, however it is captured in the manual signal assessment.

Table S3 a: Signals and life history events

Identified signal numbers	IL numbers from three sections covered by the signal**	Age at documented event	Assumed IL(s) expected to show signal*
1a	2,3	Death of mother: 11 years and 9 months	2
1b	4,5,6	No corresponding event documented	n/a
2	18,19,20	Childbirth: 29 years 8 months	19,20
No corresponding signal	n/a	Abortion: 31 years and 4 months	21,22

* Age at event minus average tooth eruption age for tooth Z_131 (canine from mandible) (according to Adler, 1967): 9.81 years

** Irregular ILs from section 1, section 2, section 3 from the respective signal are summarized.

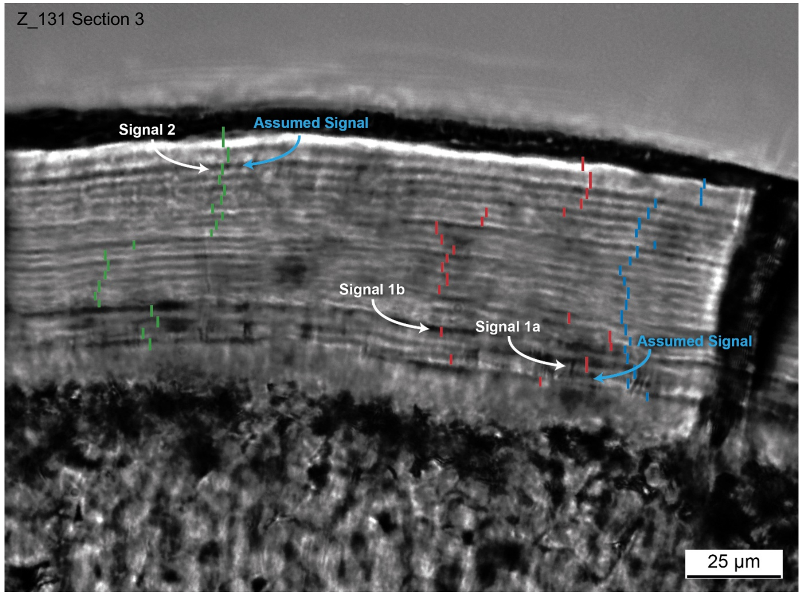

Fig. S3 a. Image of section 3 of tooth Z_131 with identified signals and in blue assumed signals indicating where signals of the documented pregnancy and the death of the mother are expected to appear (the images of the other two sections are not shown).

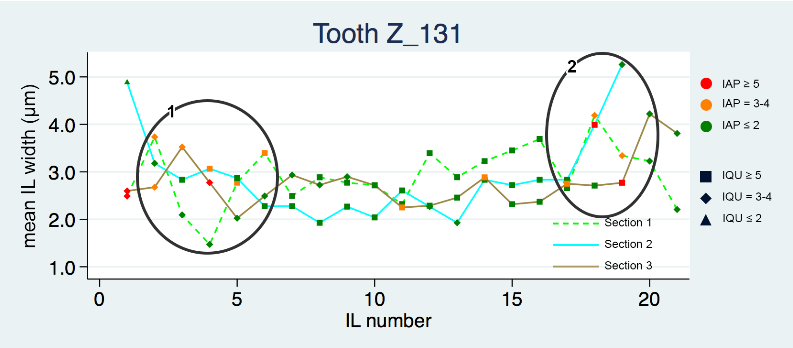

Fig. S3 b: Incremental line width growth curves of the three sections of tooth Z_131 with identified signals. Each graph describes the measurement of one section. Sub-signals are not displayed in the graphic.

Interpretation

Overall, this example shows a good overlap between signals and documented life history events. Signal 2 is clearly visible corresponding with the pregnancy in the 30st year of life. The pregnancy which ended in an abortion in the 4th month of pregnancy before the patient died, did not leave an observable signal. The last ILs at the outer cementum border have usually a lower quality (Mani-Caplazi et al., 2019) and irregularities are more difficult to identify at the border. This may explain why this abortion has not been captured as signal. Another

interesting observation is that signal 1a coincides with the death of the mother in the 12th year of life. This is an example that other life events may influence the cementogenesis. The Incremental line (IL) width growth curves have been included to illustrate the across sections signal identification with the two signals 1 and 2 clearly visible across sections.

Case Z_436 (archaeological tooth)

Name: Maria Eva Kalchschmidt

Birth date: 26.11.1802 documented in (Nussbaumer & Schlenker 2001)

Death date: 20.08.1851 (death is documented in the death register in the state archives: StABS Spital AA 2.3a Sterbe- und Beerdigungsregister 1864-1877), cause of death is documented in the medical record: lung gangrene in the state archives (StABS Spital V 30.12, Fol. 475).

Documented childbirths

- Childbirth: 24.01.1824 (illegitimate pregnancy): documented in (Nussbaumer & Schlenker 2001)
- Childbirth: 03.10.1825 (illegitimate pregnancy): documented in (Nussbaumer & Schlenker 2001)
- Childbirth: 09.03.1829 (illegitimate pregnancy): documented in (Nussbaumer & Schlenker 2001)
- Childbirth: 30.05.1831 (illegitimate pregnancy): documented in (Nussbaumer & Schlenker 2001)
- Childbirth: 07.12.1834 (illegitimate pregnancy): documented in (Nussbaumer & Schlenker 2001)
- Childbirth: 03.07.1836 (illegitimate pregnancy): documented in (Nussbaumer & Schlenker 2001)
- Childbirth: 27.06.1845 (legitimate pregnancy: married at the time of birth) documented in the church archive archived in the state archives (StABS Kirchenarchiv CC 11).

Table S3 b: Signals and life history events

**Identified signal numbers**	**IL numbers from three sections covered by the signal****	**Identified signal numbers: reverse numbering*****	**IL numbers from three sections covered by the signal*: reverse numbering*****	**Age at documented event**	**Assumed IL(s) expected to show signal***	**Assumed IL(s) expected to show signal*: reverse numbering**
1	2	-5	-33,-36	No corresponding event documented	n/a	n/a
2	10,11,12	-4	-20,-21,-23, -24,-25,-26,-27	Childbirth: 21 years and 2 months	11,12	-27,-28
3a	13,14,15	-3b	-17,-18,-20,-21, -22,-23,-24,-25	Childbirth: 22 years and 10 months	13,14	-25,-26
3b	15,17	-3a	-18,-23	Childbirth: 26 years 3 months	16,17	-22,-23
No corresponding signal	n/a	No corresponding signal	n/a	Childbirth: 28 years 6 months	18,19	-20,-21
4	20,21	-2	-14,-15,-17	Childbirth: 32 years 1 month	22,23	-16,-17
5a	20,25,29	-1b	-9,-10,-11	Childbirth: 33 years 7 months	23,24	-15,-16
5b	25,27,28,29,31	-1a	-6,-7,-8	Childbirth: 42 years 7 months	32,33	-6,-7

* Age at event minus average tooth eruption age for tooth Z_436 (canine from mandible) (according to Adler, 1967): 9.81 years.
** Irregular ILs from section 1, section 2, section 3 from the respective signal are summarized.
*** Reverse numbering starting with the last IL of the original order numbered as -1

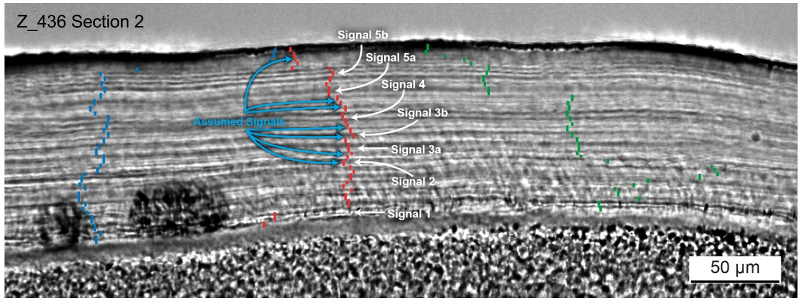

Fig. S3 c: Image of section 2 of tooth Z_436 with identified signals and assumed signals in blue indicating where signals of the documented pregnancies are expected to appear (the images of the other two sections are not shown).

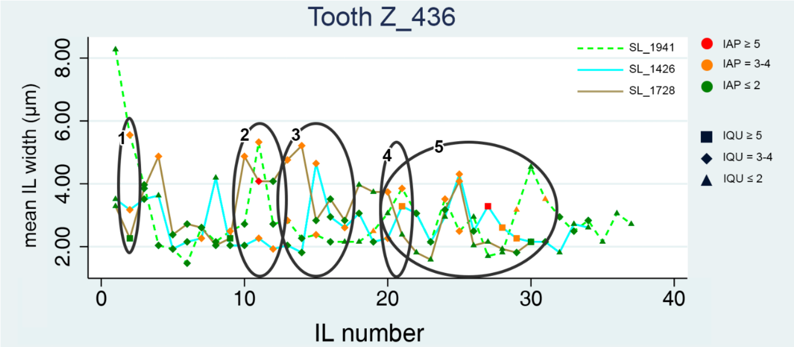

Fig. S3 d: Incremental line width growth curves of the three sections of tooth Z_436 with identified signals. Each graph describes the measurement of one section. Sub-signals are not displayed in the graphic.

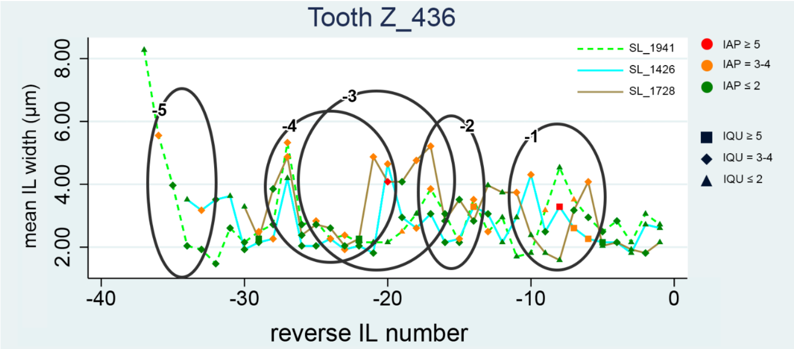

Fig. S3 e: Incremental line width growth curves of the three sections of tooth Z_436 showing the reverse numbering with identified signals. Each graph describes the measurement of one section. Sub-signals are not displayed in the graphic.

Interpretation

The tooth cementum is heterogenous with several signals and sub-signals which partially show a good concordance with documented pregnancies. However, signal allocation to the corresponding pregnancies is not straight forward. Signal 2, 3a and 3b show a relatively good concordance with documented pregnancies. Signal 3a and 3b, 4 and 5a and 5a and 5b show an overlap in signal ILs (see table), which make an allocation to pregnancies more challenging. Signal 4, 5a and 5b show a less clear concordance with documented pregnancies and some allocations to pregnancies are debatable. Pregnancy in the 29th year of life seems not to be covered by a signal. As in this tooth the total number of ILs varied substantially across the sections, we investigating also the similarity across sections based on a reverse numbering. This starts with the last IL of the original numbering in the IL width growth curves (see Fig. S3 e). As expected, this shows narrower signals near the outer border and a better overlap of pregnancy signal -1a corresponding to signal 5b in the original numbering with the last pregnancy. But, also here an overlap in several signals is present.
Overall, it seems many close pregnancies make a precise allocation more difficult in particular with increasing IL numbers. One signal in IL 2 corresponding to an age of 12 may represent another type of event and shows that other influences may have impact on the cementogenesis.

Case PRG028 (recent tooth)

Date of birth of the woman and date of extraction were taken from the medical records of dental clinics and practices in Rostock, Germany. The woman was directly asked about the number of births (including stillbirths and abortions) and the corresponding dates (Künzie und Wittwer-Backofen, 2008).
Birth date: 07.02.1943 (medical record)

Date at tooth extraction: 25.09.2001 (medical record)

Reported childbirth and abortions

- Childbirth: 15.02.1963
- Abortion: 1971 (month has not been reported)
- Abortion: 1973 (month has not been reported)

Table S3 c: Signals and life history events

**Identified signal numbers**	**IL numbers from three sections covered by the signal****	**Age at documented event**	**Assumed IL(s) expected to show signal***
1a	2,3,4,5	No corresponding event documented	n/a
1b	5,6,8	No corresponding event documented	n/a
2	11,12	Childbirth: 20 years	10,11
3a	21,23,24	Abortion: 28 years	18,19
3b	23,25	Abortion: 30 years	20,21
4	36,37,40,41	None	n/a

* Age at event minus average tooth eruption age for tooth PRG028 (canine from mandible) (according to Adler, 1967): 9.81 years.
** Irregular ILs from section 1, section 2, section 3 from the respective signal are summarized.

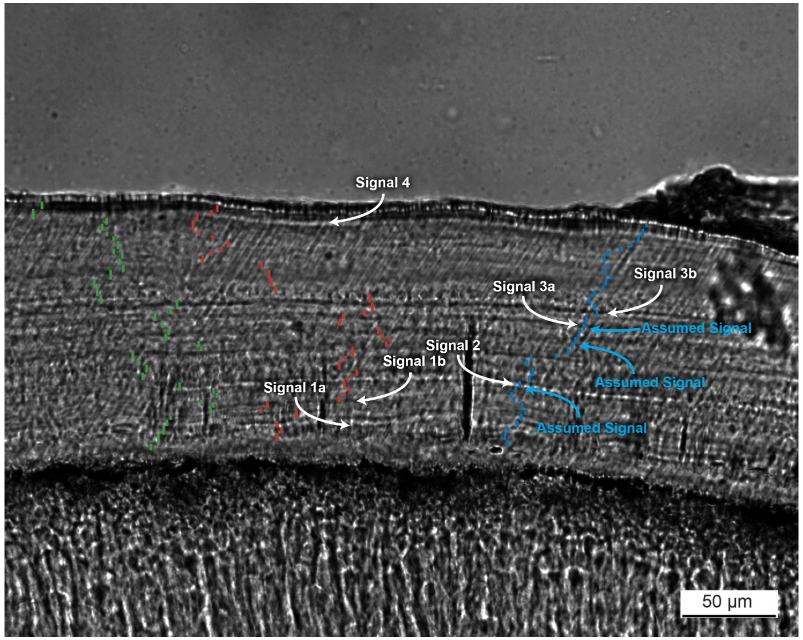

Fig. S3 f. Image of section 1 of tooth PRG028 with identified signals and assumed signals in blue indicating where signals of the documented pregnancies are expected to appear (the images of the other two sections are not shown).

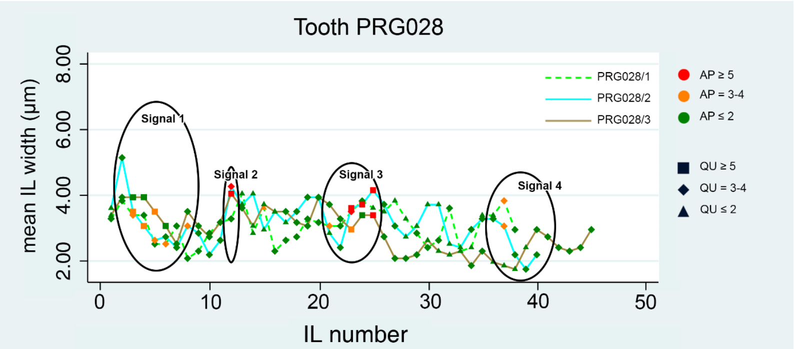

Fig. S3 g: Incremental line width growth curves of the three sections of tooth PRG028 with identified signals. Each graph describes the measurement of one section. Sub-signals are not displayed in the graphic.

Interpretation

This example shows a good overlap of the pregnancy signal and the reported childbirth but only a moderate overlap between IL numbers of signals and the ILs where the pregnancy signals of the abortions are expected. However, looking at the pattern (the ILs between the signals) the signals align well with the abortions. Kagerer

and Grupe (2001) have identified that abortions also leave qualitative markers in the tooth cementum. It is visible that the later signal 4 is covering a wider IL range. Here the alignment across sections is more challenging. The IL width growth curves have been included to illustrate the across sections signal identification with four clearly visible across sections signals. There are no life events documented which explain signal1 and signal 4 and this leaves the question as to what events other than pregnancies leave traces in the tooth cementum.

Case PRG058 (recent tooth)

This case illustrates a weaker overlap of signals and reported pregnancies.

Date of birth of the woman and date of extraction were taken from the medical records of the dental clinic in Germany. The woman was directly asked about the number of births (including stillbirths and abortions) and the corresponding dates (Künzie und Wittwer-Backofen, 2008).

Birth date: 26.06.1939 (medical record)

Date at tooth extraction: 22.11.2001 (medical record)

Reported childbirth and abortions
- Childbirth: 1959 (month has not been reported)
- Childbirth: 1962 (month has not been reported)

Table S3 d: Signals and life history events

**Identified signal numbers**	**IL numbers from three sections covered by the signal****	**Age at documented event**	**Assumed IL(s) expected to show signal***
1	7,8,9	None	n/a
2a	13,14,15	Childbirth: 20 years	11,12
2b	16,17	Childbirth: 23 years	14,15
3a	22	None	n/a
3b	24,25	None	n/a
4	26,27,30	None	n/a
5	34,37	None	n/a

* Age at event minus average tooth eruption age for tooth PRG058 (incisor from maxilla) (according to Adler, 1967): 8.18 years.
** Irregular ILs from section 1, section 2, section 3 from the respective signal are summarized.

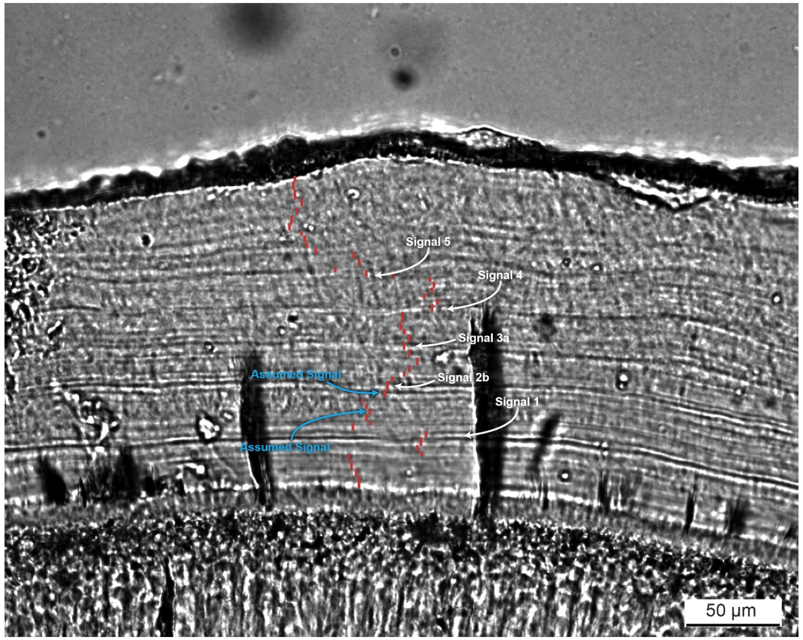

Fig. S3 h: Image of section 1 of tooth PRG058 with identified signals and assumed signals in blue indicating where signals of the documented pregnancies are expected to appear (the images of the other two sections are not shown)

Case Z_240 (archaeological tooth), example with no reported pregnancy

Name: Maria Ester Meyer

Date of birth: 10.10.1804 documented in the church record books (StABS, Kirchenarchiv CC 11,5)

Death date: 22.12.1847 (cause of death: documented in the medical record: Tuberculosis) documented in the death register in the state archives (StABS Spital AA 2.2 Sterbe- und Beerdigungsregister 1842-1864).

Documented pregnancy and one other life event

- No documented pregnancy or abortion
- Death of Maria Ester Meyer's father Johann Peter Meyer: 11.4.1821. The death is documented in the death register (StABS, Kirchenarchiv CC 16.3 Beerdigungsregister, 1812-1841).

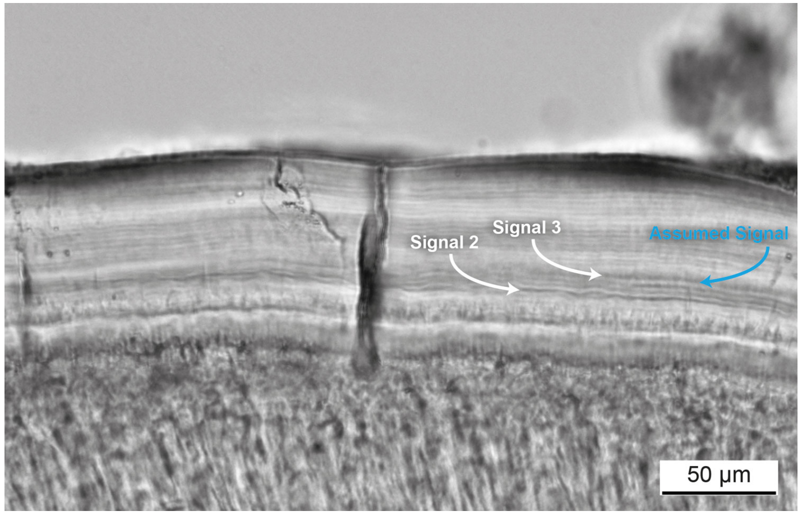

Fig. S3 i: Microscopic view of an archaeological incisor (Z_240, section 1). The tooth cementum shows a relative homogenous structure and irregular ILs are only present in the early years.

Table S3 e: Signals and life history events

**Identified signal numbers**	**IL numbers from three sections covered by the signal****	**Age at documented event**	**Assumed IL(s) expected to show signal***
1	2,3	None	n/a
2	4,5,6	None	n/a
3	8,9	Death of father: 16 years and 6 months	8,9

* Age at event minus average tooth eruption age for tooth Z_240 (incisor from mandibula) (according to Adler, 1967): 8.18 years.

Interpretation

We found that in some cases with no reported pregnancy the cementum shows a relative homogenous structure with signals only in the adolescence (Fig. S3 i). An interesting observation is that signal 3 coincides with the death of her father in the 17th year of life.

Additional references in this supplement

Church archive Sumiswald (Kirchenbücher: Sumiswald, Buch 20).

Death register (Billeter-Sammlung, GHGRB Basel).

Local family book Nussbaumer, E., Schlenker, H., 2001. Ortsfamilienbuch Buggingen (bis 1900). Hrsg.: Gemeinde Buggingen. edition gesowip. Basel, pp.147 and 232.

State archives (StABS (Staatsarchiv, Basel-Stadt): Death register, inheritance inventory, court archive, baptismal register, medical records of the community hospital Basel, church archive).
