# Supplementary material for: The concordance of signals based on irregular incremental lines in the human tooth cementum with documented pregnancies: Results from a systematic approach
Source: PLoS One. 2022 Sep 9;17(9):e0267336. doi: 10.1371/journal.pone.0267336 (PMC9462792; doi:10.1371/journal.pone.0267336)
Supplement: S4 Text — (PDF) [file pone.0267336.s004.pdf]

S4 Text: Performance of the different variants in the construction steps of the derived signals.

Since we have used a variety of variants in defining the derived signals, it is of interest to investigate, which variants are performing best, i.e. lead to the highest sensitivity relative to the prevalence. This requires quantifying for each signal variantthis difference in a way, which is comparable across different signal variants with potentially large differences in prevalence. This is not straightforward, and hence we consider three measures: 1) The increase in sensitivity, i.e. the numerical difference. 2) The odds ratio, i.e. the relative increase when both sensitivity and prevalence are expressed as odds. 3) The p-value of rejecting the null hypothesis of no difference between sensitivity and prevalence. Actually, in all these variants we replace the prevalence by the mean sensitivity under chance conditions, as there might be slight differences between these two numbers. The p-value is determined by checking how often among the 200 repetitions under chance conditions the sensitivity obtained is above the observed sensitivity.

To investigate the performance of a specific variant in the process of constructing a signal, (e.g. the variant L1 in assigning the data ranges), we take a look at the distribution of these measures when varying the other steps of this process (e.g. choosing the degree of widening or selecting a source signalvariant). The choice of the other variants is not straightforward, as the performance of one variant may depend on the choice of a variant in another step. We hence apply the following strategy: First we consider the choice of F and W separately for each variant of L. In interpreting these results, we have to take into account that variantW2 widens the age range on average by twice the amount of W1, hence in the case of similar performance W1 should be preferred, as it implies a lower signal prevalence. Second, we focus on reasonable choices of F and W identified in this first step and then consider the performance of the variants for the other steps in the construction process. We always show in the figures below the full distribution of the measures over all other variants for the other steps, and mark the median and the upper 90% percentile (for the increase in sensitivity and the odds ratio) and the lower 10% percentile (for the p-values). In the dot plots we connect the points corresponding to the same choice of the variants in the other construction steps in order to allow a judgement whether the patterns we observe for the median and the upper 90% percentile are roughly valid for any choice of variants.

All figures in this Supplementary Information depict the distribution of the increase in sensitivity, the odds ratio and the p-value over the different signal variants in dependence on the variants of one or two steps of the construction process. The odds ratios are truncated at 0.5 and 4.0, respectively. A logarithmic scale is used on the y-axis when presenting odds ratios and p-values.

Analysis of signals

In Fig. S4.1 to S4.4 we can observe that some widening of the age ranges (construction step F and W) is necessary to reach a sensitivity increase above 15% for L1, L2 and L3, and this is in particular true for L2. For L4 we cannot observe such a need. W1 and W2 imply in particular an improvement for L2, whereas F implies in particular an improvement for L1.
In the following analyses we omit for L1, L2, and L3 the choice F1, and for L2 also the choice W0. Fig. S4.5 (left side) suggests that variants L1 and L2 perform slightly better than L3 and L4, and D1 performs slight better than D2 and D3. Among the variants of the source signals, the choices S2, S8 and S9 perform best and much better than the worst performing variants S3, S4, S7, S10, and S11, which may be explained partially by the low frequency of these source signals (30, 24, 17, 50, and 35 signals). However, S12 with a similarly low frequency (22) performs distinctly better.

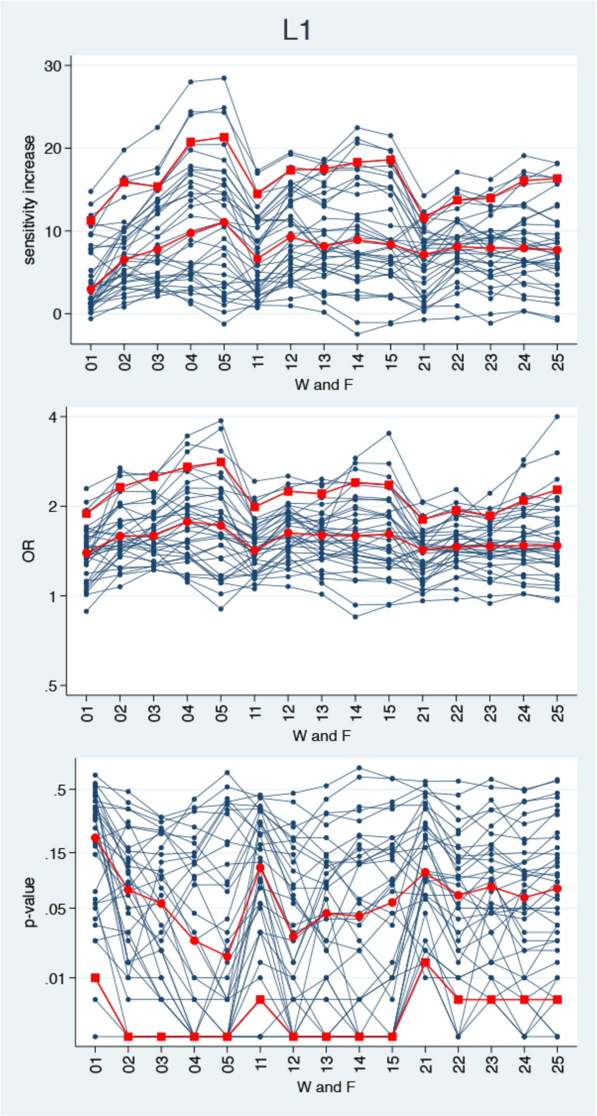

Fig S4.1: Performance Results for L1 in dependence on W and F. The x axis describes the different variants of F and W. The first digit represents the variants of W and the second one the variants of F. The median and the upper 90% percentile (for the increase in sensitivity and the odds ratio) and the lower 10% percentile (for the p-values) are marked in red.

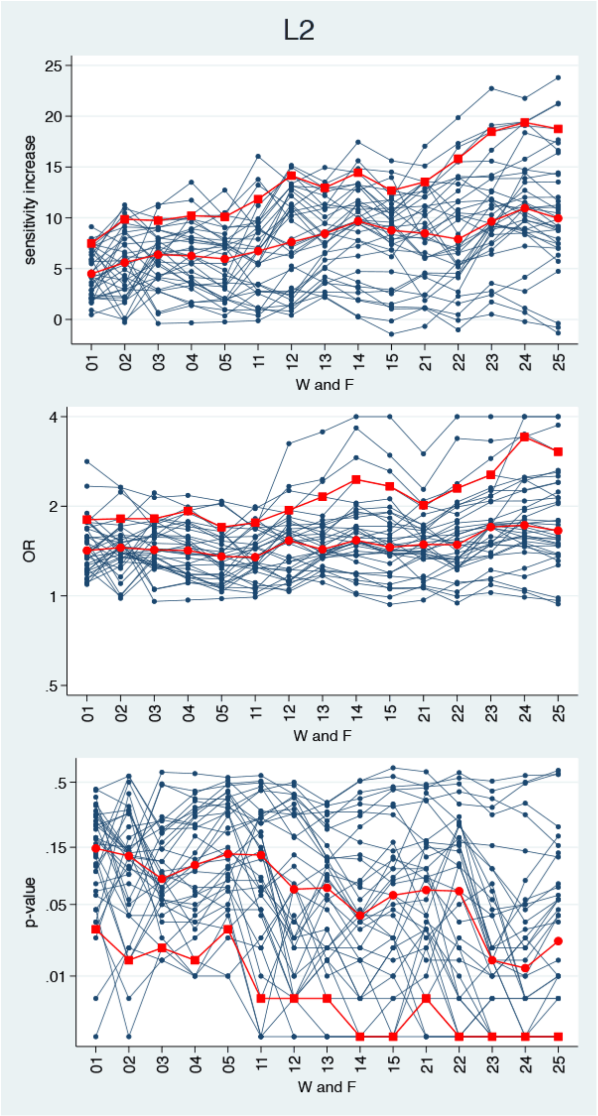

Fig S4.2: Performance Results for L2 in dependence on W and F. The x axis describes the different variants of F and W. The first digit represents the variants of W and the second one the variants of F. The median and the upper 90% percentile (for the increase in sensitivity and the odds ratio) and the lower 10% percentile (for the p-values) are marked in red.

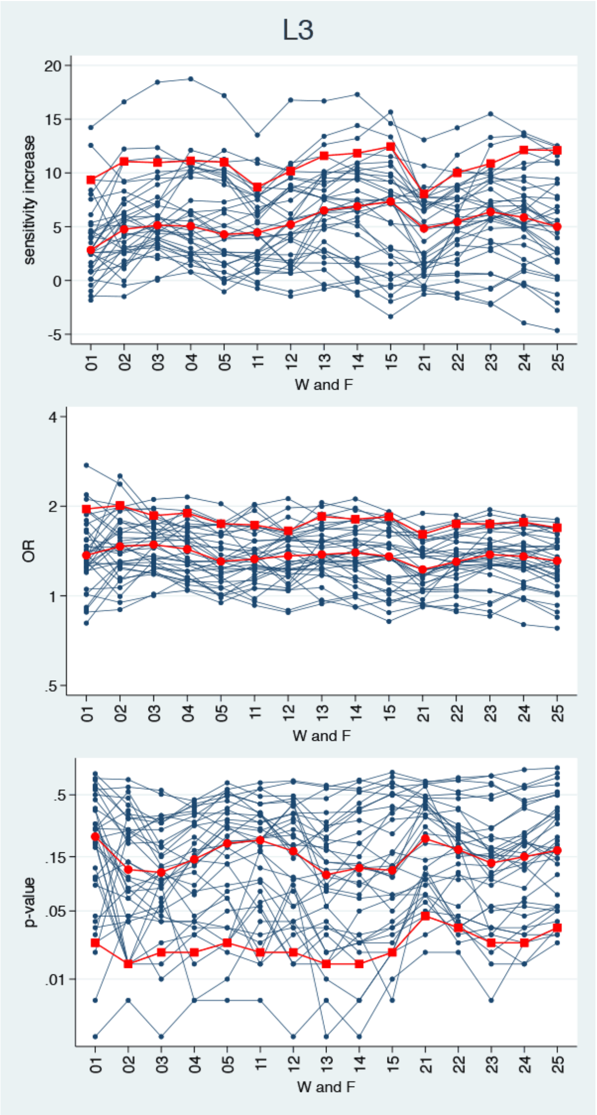

Fig S4.3: Performance Results for L3 in dependence on W and F. The x axis describes the different variants of F and W. The first digit represents the variants of W and the second one the variants of F. The median and the upper 90% percentile (for the increase in sensitivity and the odds ratio) and the lower 10% percentile (for the p-values) are marked in the reds

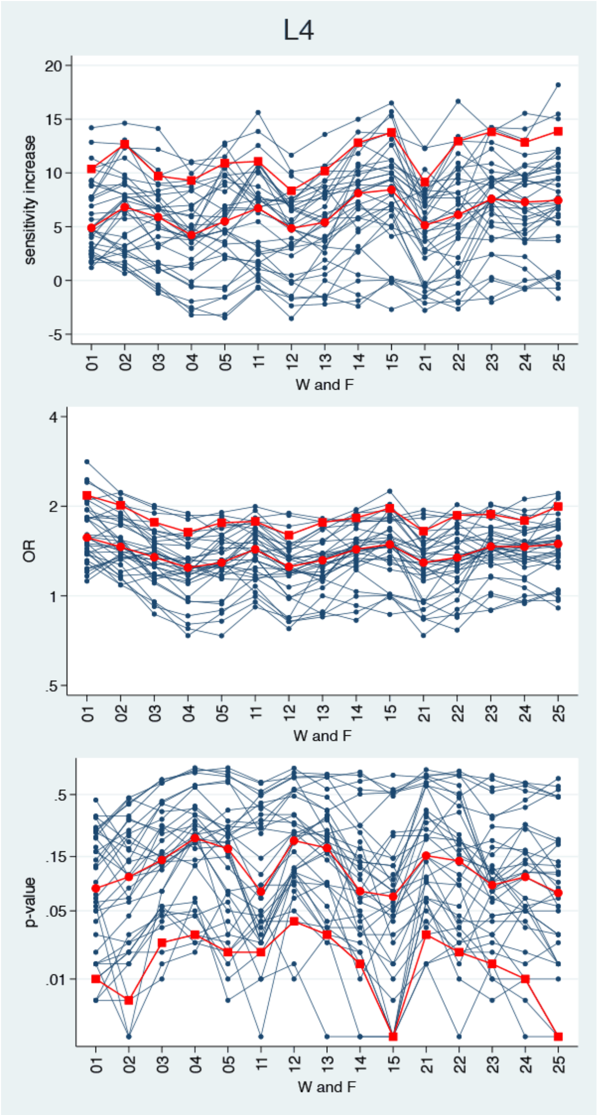

Fig S4.4: Performance Results for L4 in dependence on W and F. The x axis describes the different variants of F and W. The first digit represents the variants of W and the second one the variants of F. The median and the upper 90% percentile (for the increase in sensitivity and the odds ratio) and the lower 10% percentile (for the p-values) are marked in red.

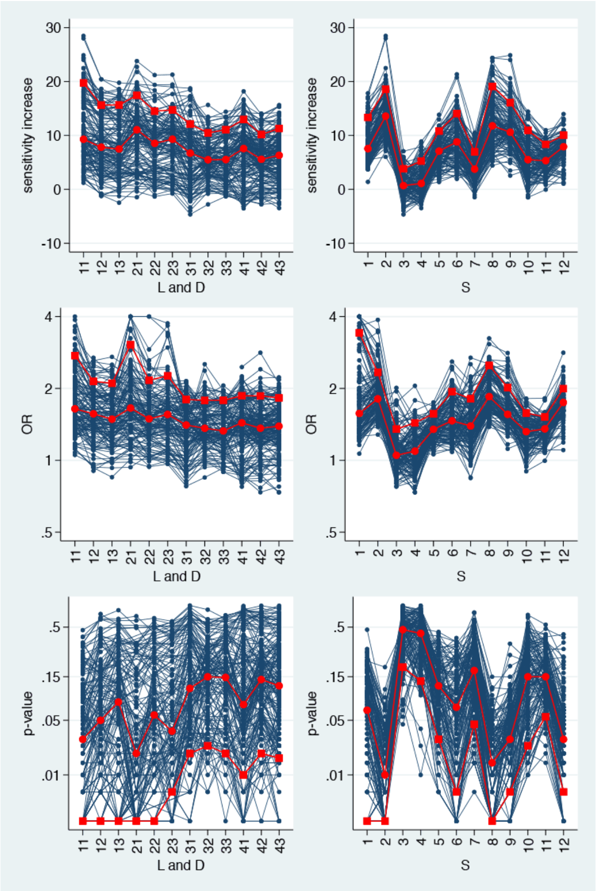

Fig S4.5: Performance Results in dependence on L and D (left side) and on S (right side).
