# Supplementary material for: The concordance of signals based on irregular incremental lines in the human tooth cementum with documented pregnancies: Results from a systematic approach
Source: PLoS One. 2022 Sep 9;17(9):e0267336. doi: 10.1371/journal.pone.0267336 (PMC9462792; doi:10.1371/journal.pone.0267336)
Supplement: S5 Text — (PDF) [file pone.0267336.s005.pdf]

S5 Text. Descriptions of datasets.

All datasets are stored as tab-delimited files.

The datasets are de-identified. The variables id and sectionid have been generated randomly and do not refer to existing identifiers. All date variables have been shifted by a random, subject-specific number in the range between -182 and +182.

S1_data.csv: Final output of signal identification

This data set includes the output from the process described in the first subsection of the Methods section.

Variable name	*Explanation*
id	Tooth/section identifier
sectionid	Section identifier
signalnumber	The number of the signal as chosen by the examiner
ILnumber	The ILs involved in the signal
signalintensity	The value of the signal intensity index
signalmatching	The value of the signal matching index

S2_data.csv: Information on each tooth / individual

Variable name	*Explanation*
id	Tooth identifier
origin	«recent» or «arch»
gender	«Male» or «Female»
birthdate	Date of birth of the adult individual
toothcode	FDI tooth code
enddate	Date of extraction or date of death
birthbyyear	Indicator that only the year of birth, but not the exact date was known

S3_data.csv: Information on each pregnancy

Variable name	*Explanation*
id	Tooth identifier
date	End date of pregnancy
byyear	Indicator that only the year of the pregnancy was known
type	«birth» or «abort»
month	The gestational month (only for abortion)
legitim	Indicator for a legitim child (only for women from the archaeological sample

S4_data.csv: Translation table for assumed date of eruption

Variable name	*Explanation*
toothcode	FDI toothcode
gender	Gender
eruptage	Assumed age of eruption

S5_data.csv: Signals according to the different definitions and their time range

Variable name	*Explanation*
id	Tooth identifier
signalnumber	The number of the signal chosen by the examiner
defS	Signal variant (S1, … , S12)
defL	Variant L
defF	Variant F
defW	Variant W
defD	Variant D
from	The start date of the time range of the signal
to	The end date of the time range of the signal

Note: The variables form and to are date variables. As these dates are computed, they are not necessarily integer values. They represent the time in days relative to January 1st, 1960.

S6_data.csv: Signal definitions and their sensitivity and prevalence

Variable name	*Explanation*
defS	Signal variant (S1, … , S12)
defL	Variant L
defF	Variant F
defW	Variant W
defD	Variant D
sens	The estimated sensitivity
prevalence	The prevalence of the signal
mean	The expected sensitivity under chance conditions
p95	The upper 5% percentile of the distribution of the estimated sensitivity under chance conditions
