# Supplementary material for: The concordance of signals based on irregular incremental lines in the human tooth cementum with documented pregnancies: Results from a systematic approach
Source: PLoS One. 2022 Sep 9;17(9):e0267336. doi: 10.1371/journal.pone.0267336 (PMC9462792; doi:10.1371/journal.pone.0267336)
Supplement: S1 Results — (PDF) [file pone.0267336.s012.pdf]

Linear regression

Number of obs	=	47
F(2, 46)	=	22.77
Prob > F	=	0.0000
R-squared	=	0.4251
Root MSE	=	1.0705

(Std. Err. adjusted for 47 clusters in id)

numsig1	Coef.	Robust Std. Err.	t	P>|t|	[95% Conf.	Interval]
truenumpreg	.0396584	.1368807	0.29	0.773	-.2358681	.3151848
N	.1331709	.0210032	6.34	0.000	.0908936	.1754482
_cons	.4691335	.4149553	1.13	0.264	-.3661282	1.304395

(1) truenumpreg = 0

F(1, 46) = 0.08
Prob > F = 0.7733

Linear regression

Number of obs	=	47
F(2, 46)	=	5.47
Prob > F	=	0.0074
R-squared	=	0.2372
Root MSE	=	1.1472

(Std. Err. adjusted for 47 clusters in id)

numsig2	Coef.	Robust Std. Err.	t	P>|t|	[95% Conf.	Interval]
truenumpreg	.1932828	.1462424	1.32	0.193	-.1010879	.4876535
N	.0719433	.0255708	2.81	0.007	.0204718	.1234147
_cons	.1225847	.537204	0.23	0.821	-.9587509	1.20392

(1) truenumpreg = 0

F(1, 46) = 1.75
Prob > F = 0.1928

Linear regression

Number of obs	=	47
F(2, 46)	=	1.93
Prob > F	=	0.1572
R-squared	=	0.0707
Root MSE	=	.75307

(Std. Err. adjusted for 47 clusters in id)

numsig3	Coef.	Robust Std. Err.	t	P>|t|	[95% Conf.	Interval]
truenumpreg	.0235831	.1032174	0.23	0.820	-.1841827	.2313489
N	.0289309	.014984	1.93	0.060	-.0012303	.059092
_cons	-.0393267	.3525103	-0.11	0.912	-.7488932	.6702397

(1) truenumpreg = 0

F(1,   46) =   0.05
Prob > F =   0.8203

Linear regression

Number of obs	=	47
F(2, 46)	=	1.99
Prob > F	=	0.1482
R-squared	=	0.0974
Root MSE	=	.60332

(Std. Err. adjusted for 47 clusters in id)

numsig4	Coef.	Robust Std. Err.	t	P>|t|	[95% Conf.	Interval]
truenumpreg	-.0244279	.0412343	-0.59	0.556	-.1074282	.0585723
N	.0294372	.0150356	1.96	0.056	-.0008279	.0597024
_cons	-.0943618	.328278	-0.29	0.775	-.7551512	.5664276

(1) truenumpreg = 0

F(1,   46) =   0.35
Prob > F =   0.5565

Linear regression

Number of obs	=	47
F(2, 46)	=	3.31
Prob > F	=	0.0454
R-squared	=	0.1159
Root MSE	=	1.2392

(Std. Err. adjusted for 47 clusters in id)

numsig5	Coef.	Robust Std. Err.	t	P>|t|	[95% Conf.	Interval]
truenumpreg	.0200322	.0702972	0.28	0.777	-.1214688	.1615332
N	.0648614	.027644	2.35	0.023	.009217	.1205059
_cons	.3040564	.5148046	0.59	0.558	-.7321914	1.340304

(1) truenumpreg = 0

F(1,   46) =   0.08
Prob > F =   0.7770

```
Linear regression                               Number of obs     =         47
                                                F(2, 46)          =       4.23
                                                Prob > F          =     0.0206
                                                R-squared         =     0.1540
                                                Root MSE          =     1.2894

                                    (Std. Err. adjusted for 47 clusters in id)
------------------------------------------------------------------------------
             |               Robust
     numsig6 |      Coef.   Std. Err.      t    P>|t|     [95% Conf. Interval]
-------------+----------------------------------------------------------------
 truenumpreg |   .0959855   .1129618     0.85   0.400    -.1313947    .3233657
           N |   .0730084   .0283101     2.58   0.013     .0160231    .1299938
       _cons |   .3327332   .5386032     0.62   0.540    -.7514189    1.416885
------------------------------------------------------------------------------

 ( 1)  truenumpreg = 0

       F(  1,    46) =    0.72
            Prob > F =    0.3999

Linear regression                               Number of obs     =         47
                                                F(2, 46)          =       3.18
                                                Prob > F          =     0.0509
                                                R-squared         =     0.0985
                                                Root MSE          =     .58759

                                    (Std. Err. adjusted for 47 clusters in id)
------------------------------------------------------------------------------
             |               Robust
     numsig7 |      Coef.   Std. Err.      t    P>|t|     [95% Conf. Interval]
-------------+----------------------------------------------------------------
 truenumpreg |    .022426   .0479041     0.47   0.642    -.0739999     .118852
           N |   .0270178   .0119763     2.26   0.029     .0029106    .0511249
       _cons |  -.2718147    .216153    -1.26   0.215    -.7069081    .1632788
------------------------------------------------------------------------------

 ( 1)  truenumpreg = 0

       F(  1,    46) =    0.22
            Prob > F =    0.6419

Linear regression                               Number of obs     =         47
                                                F(2, 46)          =       5.81
                                                Prob > F          =     0.0056
                                                R-squared         =     0.1712
                                                Root MSE          =      .8446

                                    (Std. Err. adjusted for 47 clusters in id)
------------------------------------------------------------------------------
             |               Robust
     numsig8 |      Coef.   Std. Err.      t    P>|t|     [95% Conf. Interval]
-------------+----------------------------------------------------------------
 truenumpreg |   .1322964    .077537     1.71   0.095    -.0237775    .2883703
           N |   .0396505   .0169571     2.34   0.024     .0055176    .0737835
       _cons |  -.3158928    .339993    -0.93   0.358    -1.000263    .3684776
------------------------------------------------------------------------------
```

(1) truenumpreg = 0

F(1,   46) =   2.91
Prob > F =   0.0947

Linear regression

Number of obs	=	47
F(2, 46)	=	4.30
Prob > F	=	0.0194
R-squared	=	0.1042
Root MSE	=	1.2186

(Std. Err. adjusted for 47 clusters in id)

numsig9	Coef.	Robust Std. Err.	t	P>|t|	[95% Conf.	Interval]
truenumpreg	.0914078	.1301098	0.70	0.486	-.1704897	.3533054
N	.0526087	.0226054	2.33	0.024	.0071064	.098111
_cons	.427812	.4209716	1.02	0.315	-.4195598	1.275184

(1) truenumpreg = 0

F(1,   46) =   0.49
Prob > F =   0.4859

Linear regression

Number of obs	=	47
F(2, 46)	=	4.49
Prob > F	=	0.0165
R-squared	=	0.1453
Root MSE	=	.91189

(Std. Err. adjusted for 47 clusters in id)

numsig10	Coef.	Robust Std. Err.	t	P>|t|	[95% Conf.	Interval]
truenumpreg	.054524	.077295	0.71	0.484	-.1010628	.2101109
N	.0511863	.0190046	2.69	0.010	.0129319	.0894406
_cons	-.1573951	.3636499	-0.43	0.667	-.8893843	.5745941

(1) truenumpreg = 0

F(1,   46) =   0.50
Prob > F =   0.4841

Linear regression

Number of obs	=	47
F(2, 46)	=	1.05
Prob > F	=	0.3578
R-squared	=	0.0388
Root MSE	=	.76709

(Std. Err. adjusted for 47 clusters in id)

numsig11	Coef.	Robust Std. Err.	t	P>|t|	[95% Conf.	Interval]
truenumpreg	.007807	.076025	0.10	0.919	-.1452235	.1608374
N	.0222276	.0160286	1.39	0.172	-.0100363	.0544914
_cons	.242054	.3226919	0.75	0.457	-.4074912	.8915991

(1) truenumpreg = 0

F(1, 46) = 0.01
Prob > F = 0.9187

Linear regression

Number of obs	=	47
F(2, 46)	=	1.57
Prob > F	=	0.2193
R-squared	=	0.0816
Root MSE	=	.60786

(Std. Err. adjusted for 47 clusters in id)

numsig12	Coef.	Robust Std. Err.	t	P>|t|	[95% Conf.	Interval]
truenumpreg	.0581678	.0747831	0.78	0.441	-.0923629	.2086984
N	.0196637	.0145824	1.35	0.184	-.0096892	.0490166
_cons	-.0660001	.2938957	-0.22	0.823	-.6575815	.5255814

(1) truenumpreg = 0

F(1, 46) = 0.61
Prob > F = 0.4407
