# Supplementary material for: The concordance of signals based on irregular incremental lines in the human tooth cementum with documented pregnancies: Results from a systematic approach
Source: PLoS One. 2022 Sep 9;17(9):e0267336. doi: 10.1371/journal.pone.0267336 (PMC9462792; doi:10.1371/journal.pone.0267336)
Supplement: Attachment — Submitted filename: Responseletter.docx [file pone.0267336.s013.docx]

Response letter

We are very grateful to the reviewers and the academic editor for their positive and constructive comments. They allowed us to improve the manuscript further.

The points raised by the editor and the reviewers are addressed below.

When submitting your revision, we need you to address these additional requirements.

1. Please ensure that your manuscript meets PLOS ONE's style requirements, including those for file naming. The PLOS ONE style templates can be found at 
https://journals.plos.org/plosone/s/file?id=wjVg/PLOSOne_formatting_sample_main_body.pdf and 
https://journals.plos.org/plosone/s/file?id=ba62/PLOSOne_formatting_sample_title_authors_affiliations.pdf

We added the city names to the affiliations and changed the font types according to the template. We hope that the naming of the supplementary files is acceptable. We found instructions for using S1_Fig.pdf as well as for S1_fig.pdf.

2. Please amend your current ethics statement to address the following concerns:
a) Did participants provide their written or verbal informed consent to participate in this study?
b) If consent was verbal, please explain i) why written consent was not obtained, ii) how you documented participant consent, and iii) whether the ethics committees/IRB approved this consent procedure.

After consultation with PLOS One, the ethics statement was changed to

With respect to the recent sample, the participants provided informed verbal consent but the consent was not documented. There was no separate requirement for approval by an ethics committee. With respect to the archaeological sample from 19th century Switzerland, the use of the data for research purposes has been accepted by the data protection officer of the city of Basel. The data protection officer confirmed that -- according to the Swiss law -- working with such data does not require additional specific permission, institutional approval by an ethics committee, or an IRB.

3. We noted in your submission details that a portion of your manuscript may have been presented or published elsewhere.

"The manuscript is part of the first author's PhD thesis, which have been accepted by the Unversity of Basel. However, the thesis is not published. It will be made available to the public in April 2023."

Please clarify whether this [conference proceeding or publication] was peer-reviewed and formally published. If this work was previously peer-reviewed and published, in the cover letter please provide the reason that this work does not constitute dual publication and should be included in the current manuscript.

We have extended our description in the submission details in the following way:

The manuscript is part of the first author's PhD thesis, which has been accepted by the University of Basel. According to the rules of the University of Basel, a PhD thesis is peer-reviewed after submission. This submission process resulted in acceptance of the thesis by the University of Basel. However, the publication of the thesis (i.e., the step to make it publicly available at the Library of the university) can be delayed. The PhD student has chosen this option. Hence it is not yet formally published. It will be made available to the public in April 2023.

In the manuscript we added the following information

The original version of this manuscript is part of the first author's PhD thesis, which has been accepted by the University of Basel. The thesis will be made publicly available in March 2023.

4. Please include your full ethics statement in the ‘Methods’ section of your manuscript file. In your statement, please include the full name of the IRB or ethics committee who approved or waived your study, as well as whether or not you obtained informed written or verbal consent. If consent was waived for your study, please include this information in your statement as well.

The full ethics statement (see point 2 above) has been added to the Methods Section. The text in the section «Material» was adapted accordingly.

5. Please review your reference list to ensure that it is complete and correct. If you have cited papers that have been retracted, please include the rationale for doing so in the manuscript text, or remove these references and replace them with relevant current references. Any changes to the reference list should be mentioned in the rebuttal letter that accompanies your revised manuscript. If you need to cite a retracted article, indicate the article’s retracted status in the References list and also include a citation and full reference for the retraction notice.

We added the missing publication year in one publication and a doi to a publication without page numbers.

Additional Editor Comments:

Thank you for submitting your manuscript to PLOS ONE. It is my pleasure to inform you that it has been accepted for publication pending you address a few minor revisions suggested by the Reviewers. I really look forward to handling the revision and to seeing this paper published if you are able to address these comments satisfactorily. I will evaluate the revisions. The paper will not need further review.

[Note: HTML markup is below. Please do not edit.]

Reviewers' comments:

Reviewer's Responses to Questions

**Comments to the Author**

1. Is the manuscript technically sound, and do the data support the conclusions?

The manuscript must describe a technically sound piece of scientific research with data that supports the conclusions. Experiments must have been conducted rigorously, with appropriate controls, replication, and sample sizes. The conclusions must be drawn appropriately based on the data presented.

Reviewer #1: Yes

Reviewer #2: Partly

2. Has the statistical analysis been performed appropriately and rigorously?

Reviewer #1: Yes

Reviewer #2: Yes

3. Have the authors made all data underlying the findings in their manuscript fully available?

The [PLOS Data policy](http://www.plosone.org/static/policies.action#sharing) requires authors to make all data underlying the findings described in their manuscript fully available without restriction, with rare exception (please refer to the Data Availability Statement in the manuscript PDF file). The data should be provided as part of the manuscript or its supporting information, or deposited to a public repository. For example, in addition to summary statistics, the data points behind means, medians and variance measures should be available. If there are restrictions on publicly sharing data—e.g. participant privacy or use of data from a third party—those must be specified.

Reviewer #1: Yes

Reviewer #2: Yes

4. Is the manuscript presented in an intelligible fashion and written in standard English?

PLOS ONE does not copyedit accepted manuscripts, so the language in submitted articles must be clear, correct, and unambiguous. Any typographical or grammatical errors should be corrected at revision, so please note any specific errors here.

Reviewer #1: Yes

Reviewer #2: Yes

5. Review Comments to the Author

Please use the space provided to explain your answers to the questions above. You may also include additional comments for the author, including concerns about dual publication, research ethics, or publication ethics. (Please upload your review as an attachment if it exceeds 20,000 characters)

Reviewer #1: A sound and painstaking study based on unique material, dedicated to check "reverse" hypothesis: if pregnancies leave detectable traces on dental cementum incremental lines, maybe it is possible to determine past pregnancies from incremental lines? Authors show that notwithstanding all efforts relation absent but poor and insufficient for use in e.g.paleodemography (or forensic identification).
Minor comments: more details on protocol how teeth sections were performed and tools used for visualisation (now only reference given - please check the publication date, it is missing). I would suggest also more technical details from the main text (especially Methods section) move to Supplements

Details on the protocol how teeth sections were performed and on tools used for visualization are now added as an extra paragraph in the Section “Material”. The paragraph reads

“The teeth had been embedded in synthetic resin and up to five consecutive cross sections per tooth from the apical part of the middle third of the tooth root towards the crown were prepared applying the technique described in [17]. Then sections (typically three) with the best IL quality were selected according to criteria described in the supplementary information of [24]. These selected sections were then examined by transmission light microscopy in ×25 to ×400 magnification and areas with good IL presentation were scanned using a digital camera.”

In addition, the missing publication date was added.

We do not follow the suggestion of the reviewer to move material from the Methods section to the Supplement. In our opinion, all information provided in the Methods part is essential in order to be able to follow the Results section.

Reviewer #2: Investigation of cementum variation to identify pregnancy is a promising are of interest, yet a very complicated one considering all the potential confounding factors. This particular team of researchers has been at the forefront of cementum studies for over 20 years, and their contribution is always welcome. However, the methodology developed in their previous papers to define IL, and this follow up seem rather cumbersome, maybe unnecessarily so. What they define as a standardized, reproducible method, is as far as I am concerned, not efficient enough, and simply too cumbersome to be of any really use, especially in paleodemography. However, I welcome the rigorous approach and systematic review of limits of the study that does contribute to move research forward. However, I am a bit surprised that they have disregarded entirely a whole body of evidence (Newham and colleagues 2021; 2022; Newham and Naji 2022) that has already proven to be potentially more informative (but not necessarily applicable at a wider scale) regarding the influence of pregnancies on cementum variation. This is disappointing considering that the one of the authors (UWB) has contributed to one edited volume covering the topic (Naji, Rendu and Gourichon 2022).

We took a look at the additional references, which were partially published in parallel to the manuscript. One of these references gives an excellent overview about the state of the art in this field and we added it to the list of references on previous work in this area. In addition, we found the paper on a semiautomatic approach very interesting and integrated a reference to it in the discussion.

Finally, my main criticism would be that the combination of the uncertainty linked to the authors original IL definition, combined with the potential biological variability of cementum growth will probably never result in a precise identification method dedicated to pregnancies. Potentially, any major growth alteration variable might have a confounding effect on a visual inspection of a AEFC cementum semi-thin section. Using a male control group would have been a powerful argument to justify (or discard) the original IL method in terms of picking out signals.

We agree with the author that our work may rise the question whether there is a general limitation in detecting pregnancies in the tooth cementum. We added a paragraph on this combining it with the perspective of using semi-automatic approaches. The paragraph reads

“However, our investigation also suggests that even with a structured approach, the manual visual detection of irregularities remains challenging and potentially insufficient. It might be a promising alternative to use semi-automatic approaches in order to achieve an objective assessment with limited resources. Such approaches have been suggested for counting ILs (e.g. [33]), but their extension to detecting irregularities is still lacking.”

We agree with the author that a male control group would be of interest as soon as a rule for identifying pregnancies has been established. However, this paper is still about the process of constructing this rule, and actually we failed to come to a suggestion for one clear rule. Hence at this stage of the research process, a control group is of limited value.

Nevertheless, as stated previously, any contribution at that level that moves the debate forward is welcome.

6. PLOS authors have the option to publish the peer review history of their article ([what does this mean?](https://journals.plos.org/plosone/s/editorial-and-peer-review-process#loc-peer-review-history)). If published, this will include your full peer review and any attached files.


If you choose “no”, your identity will remain anonymous but your review may still be made public.


**Do you want your identity to be public for this peer review?** For information about this choice, including consent withdrawal, please see our [Privacy Policy](https://www.plos.org/privacy-policy).

Reviewer #1: No

Reviewer #2: No

[NOTE: If reviewer comments were submitted as an attachment file, they will be attached to this email and accessible via the submission site. Please log into your account, locate the manuscript record, and check for the action link "View Attachments". If this link does not appear, there are no attachment files.]

While revising your submission, please upload your figure files to the Preflight Analysis and Conversion Engine (PACE) digital diagnostic tool, <https://pacev2.apexcovantage.com/>. PACE helps ensure that figures meet PLOS requirements. To use PACE, you must first register as a user. Registration is free. Then, login and navigate to the UPLOAD tab, where you will find detailed instructions on how to use the tool. If you encounter any issues or have any questions when using PACE, please email PLOS at [figures@plos.org](mailto:figures@plos.org). Please note that Supporting Information files do not need this step.
